# Rapid Identification of Cortical Motor Areas in Rodents by High-Frequency Automatic Cortical Stimulation and Novel Motor Threshold Algorithm

**DOI:** 10.3389/fnins.2017.00580

**Published:** 2017-10-17

**Authors:** Mitsuaki Takemi, Elisa Castagnola, Alberto Ansaldo, Davide Ricci, Luciano Fadiga, Miki Taoka, Atsushi Iriki, Junichi Ushiba

**Affiliations:** ^1^School of Fundamental Science and Technology, Graduate School of Science and Technology, Keio University, Kanagawa, Japan; ^2^Laboratory for Symbolic Cognitive Development, RIKEN Brain Science Institute, Saitama, Japan; ^3^Center for Translational Neurophysiology of Speech and Communication, Istituto Italiano di Tecnologia, Ferrara, Italy; ^4^Graphene Labs, Istituto Italiano di Tecnologia, Genova, Italy; ^5^Section of Human Physiology, University of Ferrara, Ferrara, Italy; ^6^Department of Biosciences and Informatics, Faculty of Science and Technology, Keio University, Kanagawa, Japan; ^7^Keio Institute of Pure and Applied Sciences, Keio University, Kanagawa, Japan

**Keywords:** cortical stimulation mapping, cortical surface stimulation, direct electrical stimulation, electrocorticographic (ECoG), epidural cortical stimulation, motor mapping, motor representation

## Abstract

Cortical stimulation mapping is a valuable tool to test the functional organization of the motor cortex in both basic neurophysiology (e.g., elucidating the process of motor plasticity) and clinical practice (e.g., before resecting brain tumors involving the motor cortex). However, compilation of motor maps based on the motor threshold (MT) requires a large number of cortical stimulations and is therefore time consuming. Shortening the time for mapping may reduce stress on the subjects and unveil short-term plasticity mechanisms. In this study, we aimed to establish a cortical stimulation mapping procedure in which the time needed to identify a motor area is reduced to the order of minutes without compromising reliability. We developed an automatic motor mapping system that applies epidural cortical surface stimulations (CSSs) through one-by-one of 32 micro-electrocorticographic electrodes while examining the muscles represented in a cortical region. The next stimulus intensity was selected according to previously evoked electromyographic responses in a closed-loop fashion. CSS was repeated at 4 Hz and electromyographic responses were submitted to a newly proposed algorithm estimating the MT with smaller number of stimuli with respect to traditional approaches. The results showed that in all tested rats (*n* = 12) the motor area maps identified by our novel mapping procedure (novel MT algorithm and 4-Hz CSS) significantly correlated with the maps achieved by the conventional MT algorithm with 1-Hz CSS. The reliability of the both mapping methods was very high (intraclass correlation coefficients ≧0.8), while the time needed for the mapping was one-twelfth shorter with the novel method. Furthermore, the motor maps assessed by intracortical microstimulation and the novel CSS mapping procedure in two rats were compared and were also significantly correlated. Our novel mapping procedure that determined a cortical motor area within a few minutes could help to study the functional significance of short-term plasticity in motor learning and recovery from brain injuries. Besides this advantage, particularly in the case of human patients or experimental animals that are less trained to remain at rest, shorter mapping time is physically and mentally less demanding and might allow the evaluation of motor maps in awake individuals as well.

## Introduction

Primary motor cortex (M1) is characterized by a somatotopic organization: different body parts are represented in different locations with some extent of overlap. This organization, called motor map, changes its spatial characteristics over time, such as during or after motor learning (Pascual-Leone et al., 1995; Nudo et al., 1996a; Kleim et al., 1998, 2002; Plautz et al., 2000; Molina-Luna et al., 2008), by rehabilitation after stroke (Nudo and Milliken, 1996; Nudo et al., 1996b) and following amputation (Karl et al., 2001; Mercier et al., 2006). Nowadays a variety of techniques are utilized to study the motor map (Sejnowski et al., 2014). Cortical stimulation mapping is widely accepted as a method for functional parcellation of the motor cortex, from basic experiments to clinical applications. In the mapping, electric currents invasively applied to the cortex or electromagnetic pulses applied through the scalp (transcranial magnetic stimulation, TMS) stimulate a limited brain area and evoke muscle activity, which can be quantified by electromyography (EMG). A motor map is created using a dataset consisting of correlations between stimulated neurons (i.e., a brain area) and behavioral output (i.e., muscle twitches) (Gioanni and Lamarche, 1985; Nudo et al., 1990, 1996a; Huntley, 1997).

Cortical surface stimulation (CSS), in which electric currents are applied using surface electrodes placed over the dura or pia mater, allows to balance invasiveness of the stimulation and the spatial resolution (in the order of mm^2^; Slutzky et al., 2010), compared with other stimulation mapping techniques. For example, intracortical microstimulation (ICMS; Asanuma and Sakata, 1967) that uses needle electrodes inserted into specific cortical layers for stimulation could cause damage to the neurons (Donoghue and Wise, 1982; Neafsey et al., 1986; Rajan et al., 2015) and thus hampers the assessment of cortical plasticity in longitudinal studies. TMS is a non-invasive mapping technique utilized in many human studies, but the spatial resolution is assumed to be in the order of cm^2^ (Brasil-Neto et al., 1992). This resolution is too coarse for several experimental animals, such as rodents and non-human primates, which limits its use as a tool for preoperative mapping in neurological patients (Picht et al., 2013; Krieg et al., 2014).

CSS has great potential as a tool to investigate motor maps in animal experiments and is regarded as the gold standard for human preoperative mapping. However, data acquisition of a single map is generally time consuming (van de Ruit et al., 2015), limiting the evaluation of short-term plastic changes. Given the findings that M1 neurons change their muscle representation depending on arm postures (Kakei et al., 1999) and immediately after nerve injury (Sanes et al., 1988), the reduction of mapping time could enable to elucidate dynamic properties of M1 at the level of somatotopic organization. Further, in clinical practice lengthy mapping protocols are mentally and physically demanding for patients. As a result, most studies have shortened the mapping time by limiting map accuracy and compromising on map reliability.

The purpose of this study is to establish a reliable CSS mapping method in which the time needed to create the motor map is reduced to the order of a few minutes. Our approach (called “novel mapping”) to achieve this goal can be summarized as follows: (1) we proposed a new criterion that enables the estimation of the motor threshold (MT) with a smaller number of stimuli. The MT is defined as the stimulus intensity that elicits a detectable EMG response with 50% probability. This is a measure in which motor maps are generally created and known as one of the most reliable measures of corticospinal excitability (Ngomo et al., 2012). In clinical practice and TMS studies on humans, the MT is mostly defined as the lowest cortical stimulus intensity required to elicit a certain EMG amplitude in 5 of 10 stimuli (Rossini et al., 1994). Such an estimation of the MT based on the relative frequency is known to be independent of the probabilistic nature of the nervous system (Groppa et al., 2012). This means that a single relative frequency criterion can be utilized in humans, non-human primates and rodents, which could have different excitatory properties in the nervous system. However, the method proposed by Rossini et al. (1994) is rather time consuming and requires a relatively high number of stimuli (Tranulis et al., 2006). (2) Second, we developed a motor mapping system that automatically changes the stimulating electrode and modulates the intensity on the basis of a pre-programmed algorithm. In this study, the electrodes were randomly selected, one-by-one for each stimulus. The stimulus intensity was changed according to the previously evoked EMG potentials, based on a rule for MT estimation in a closed-loop fashion. (3) Third, for further reduction of mapping time, CSS was repeated at 4 Hz in combination with the novel algorithm for relative frequency MT estimation.

As a control, the conventional MT estimation algorithm (Rossini et al., 1994) was used with CSS repeated at 1 Hz, which is the most used stimulus frequency for motor mapping in animals (Nudo et al., 1990; Plautz et al., 2000; Frost et al., 2013; Rajan et al., 2015) and considered as the maximum stimulus frequency capable of reliably mapping the motor cortex in humans (van de Ruit et al., 2015). In the present study this method was referred to as “conventional mapping.” As a proof of concept, we validated the novel mapping in comparison with the conventional mapping using 12 adult rats. Moreover, we performed motor mappings using ICMS in two of the 12 rats and compared the motor maps obtained by the novel CSS to those obtained with ICMS mappings for further validation.

## Materials and methods

### Animals and surgical procedure

Twelve male Wister rats (11–15 weeks, 360–430 g) were used in the present study. All interventions and animal care procedures were carried out in accordance with the Laboratory Animal Welfare Act and The Guide for the Care and Use of Laboratory Animals (National Institutes of Health, Bethesda, MD) and were approved by the Institutional Animal Research Committee at RIKEN (IRB approval number H24-2-228). All surgeries were performed under medetomidine/midazolam/butorphanol anesthesia (0.15/2.0/5.0 mg/kg intraperitoneally). After anesthesia, enamel insulated copper wires (160 μm diameter) were inserted into the left extensor carpi radialis (ECR) and the left soleus muscles for EMG recordings and in subcutaneous neck tissue for the recording ground. Impedance of all EMG channels was kept below 20 kΩ throughout the whole experiment. Anesthetized rats were then secured in ear bars of a stereotactic frame (SR-5R; Narishige, Tokyo, Japan). Lidocaine gel was put on the ear bar to prevent ear pain. Body temperature was maintained with a heating pad. A craniotomy of 9 × 5 mm^2^ was then performed by drilling above the forelimb and hindlimb regions of the sensorimotor cortex (coordinates relative to bregma: 4.5 mm caudal, 4.5 mm rostral and 0.5–5.5 mm lateral; Gioanni and Lamarche, 1985) to expose the to-be-stimulated cortex. After the experiment, the animals were euthanized with an overdose of pentobarbital.

### Automatic motor mapping system

**Figure 1:**
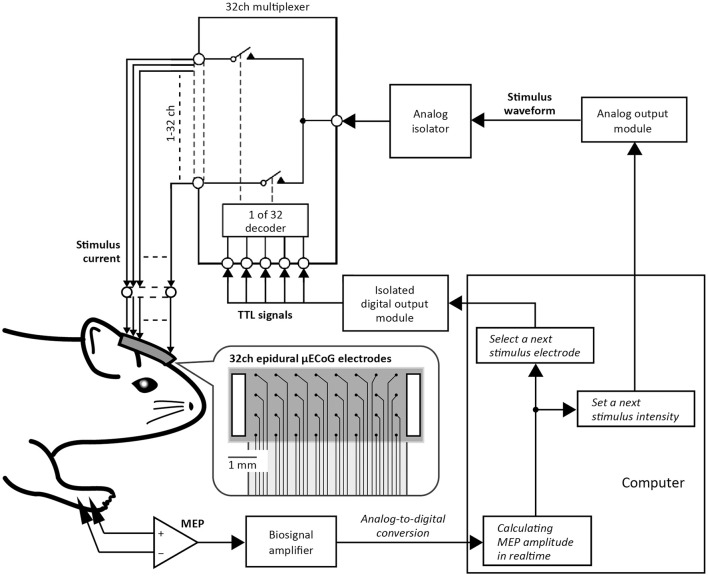
Automatic motor mapping system. Stimulus current was applied epidurally through one of the 32 micro-electrocorticographic (μECoG) electrodes placed on the rat motor cortex. In the picture of the 32ch ECoG electrode sheet, black dots represent stimulus electrodes and white boxes represent stimulus grounds. Individual electrodes for cortical stimulation were selected by a 32ch multiplexer that was controlled using transistor-transistor logic (TTL) signals sent from the isolated digital output module. The stimulus waveform (e.g., duration, polarity, and intensity) was regulated by a signal sent from the analog output module. Motor evoked potential (MEP) was then stored on a computer. Thereafter, the implemented algorithm selected the μECoG electrode and the intensity value of the next stimulation, depending on the previous MEP amplitude.

Figure 1 shows a schematic diagram of the automatic motor mapping system. Electrical cortical stimulation was applied through a micro-electrocorticographic (μECoG) array of 32 channels coated with a poly (3,4-ethylenedioxythiophene)-carbon nanotubes (PEDOT-CNT) composite (Castagnola et al., 2013). The 32 electrodes (100 μm in diameter) were arranged on a matrix with a pitch of 700 μm and an area of 5.0 × 2.2 mm^2^. The sheet size is 6.8 × 2.6 mm^2^, and stimulus grounds (0.5 × 2.2 mm^2^) are placed at the short side edge of the array. The μECoG array was put on the dura mater of the right primary sensorimotor cortex. To apply an electrical stimulation through a single arbitrary channel from among 32 electrodes, stimulus current was diverged by the multiplexer (ADG406; Analog Devices, Norwood, MA). The multiplexer connected a single μECoG electrode to the analog isolator output (SS-203J; Nihon Koden, Tokyo, Japan). During the stimulation, the pattern of connection between the stimulating unit and the individual electrodes was switched by transistor-transistor-logic level digital signals transferred from the digital output module, which was isolated from ground and the other modules (NI9403; National Instruments, Austin, TX). Stimulus current (i.e., the analog isolator output) was controlled by the signal sent from the analog output module (NI PCIe-6321; National Instruments). In the present study, the stimulus consisted of 10 500-μs biphasic pulses (cathode first) delivered at 1000 Hz and the maximum stimulator output (MSO) was adjusted to 1.0 mA. Stimulus intensity can be modulated from 20 to 100% of MSO with 5% steps. EMG signals recorded from the ECR and soleus muscles were band-pass filtered (1–2000 Hz with 2nd order Butterworth) and digitized at 4800 Hz using a biosignal amplifier (g.USBamp; g.tec medical engineering, Graz, Austria). The amplitude of EMG activity evoked by the stimulation, which is referred to as motor evoked potential (MEP), was calculated online and was used to select a μECoG electrode and an intensity for the next stimulation using in-house developed scripts (see Experimental Procedure in Motor Mapping by Epidural Cortical Stimulation section) based on the history of prior MEPs in a closed-loop fashion.

### Motor mapping by epidural cortical stimulation

#### Experimental procedure

**Figure 2:**
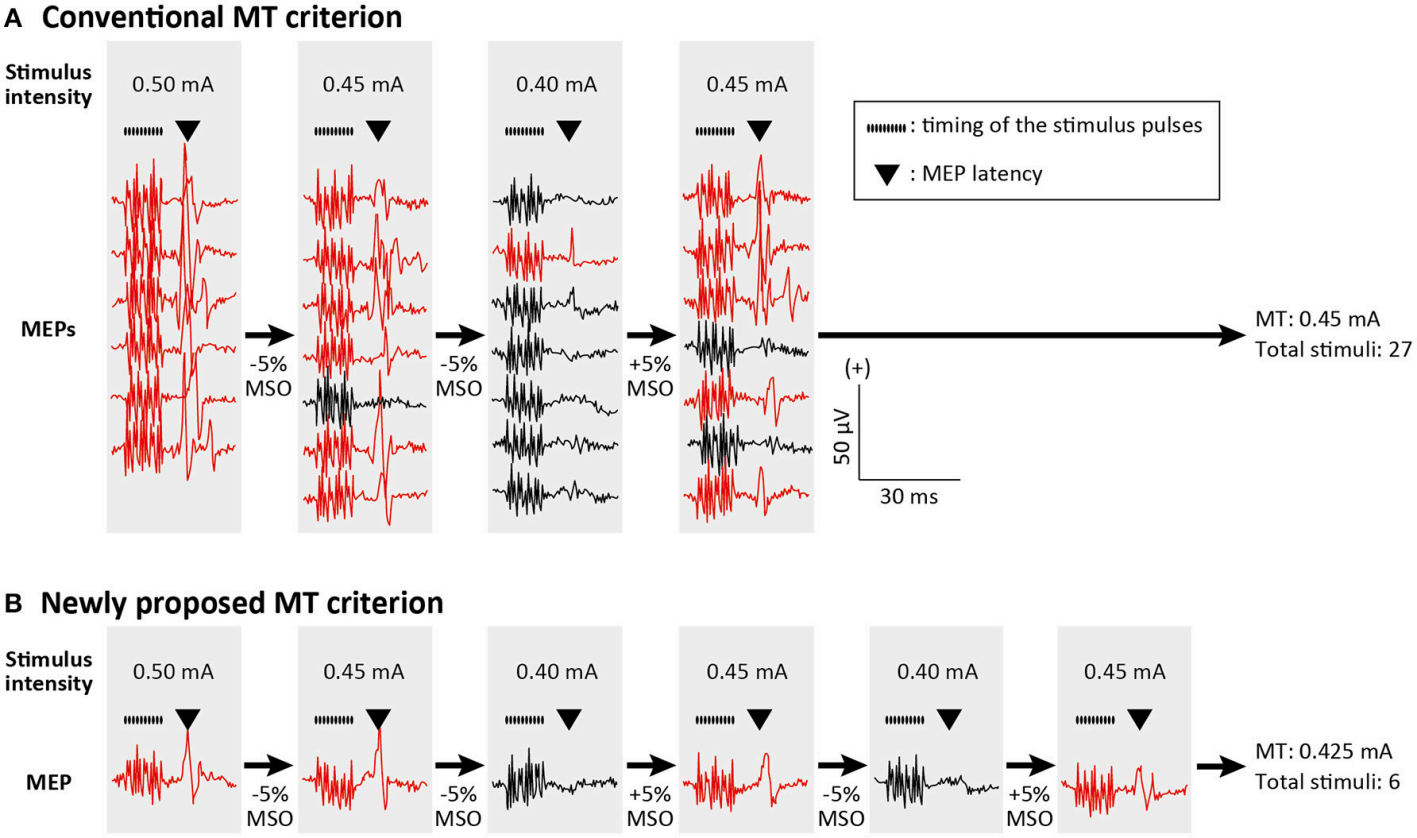
Conventional and newly proposed criteria for motor threshold (MT) estimation. MT estimation procedure in a single stimulating channel is depicted. MEPs greater and smaller than the predetermined amplitude are illustrated with red and black traces, respectively. Train of the black dots and triangle above the MEP traces indicate the timing of stimulus pulses and MEP latencies, respectively. **(A)** According to the conventional criterion, MT was defined as the lowest stimulus intensity capable of evoking MEPs greater than the predetermined amplitude (e.g., 50 μV) in at least five of 10 consecutive trials (in the figure only 7 trials are shown). If more than five significant or non-significant MEPs had been observed while fewer than 10 stimuli were delivered, the next stimulus intensity was decreased or increased by 5% of the maximum stimulator output (MSO). **(B)** In the newly proposed criterion, the next stimulus intensity was decreased or increased by 5% of the MSO if the actual MEP was larger or smaller than the predetermined amplitude, respectively. As the MT we defined the mean stimulus intensity of the last two stimuli if the intensity changes in the last five stimuli were within ±5% of the MSO.

In the present study, the cortical motor map was assessed using two different MT criteria. The first criterion (Conventional MT; Figure 2A) was the lowest stimulus intensity capable of evoking MEPs greater than the predetermined amplitude in at least five of 10 consecutive trials (Rossini et al., 1994). Here, if more than five significant or non-significant MEPs had been observed while fewer than 10 stimuli were delivered, the automatic mapping system decreased or increased the next stimulus intensity by 5% of the MSO, respectively. The second criterion was designed for estimating the MT with a smaller number of stimuli (Figure 2B), as is classical in the threshold tracking of human peripheral nerve excitability (Bostock et al., 1998). In the present study, we adapted this classic method for cortical MT estimation. The next stimulus intensity was shifted according to a single MEP response elicited by the previous stimulus. If the present MEP was larger or smaller than the predetermined amplitude, the next stimulus intensity decreased or increased by 5% of the MSO, respectively. The MT was defined as the mean stimulus intensity of the last two stimuli if the intensity changes in the last five stimuli were within ±5% of the MSO. This novel algorithm is implemented as a MATLAB function and can be downloaded from a website (https://www.mathworks.com/matlabcentral/fileexchange/64280).

The mapping procedure was started with an intensity of 50% MSO (0.5 mA). A stimulus current was randomly applied to one of the 32 electrodes. The MT of each electrode was estimated separately, and the electrode at which the MT value had been defined was no longer given stimulation. In addition, the electrode where the significant MEP was not evoked at 100% MSO (1.0 mA) was designated as nonresponsive. The amplitude of significant MEP response was determined prior to the mapping procedure in each rat. This depended on the noise level of the EMG signals and fixed at the minimum amplitude at which the MEP can be distinguished from noise (Rossini et al., 1994). The amplitude ranged between 30–50 μV in the ECR muscle and 25–40 μV in the soleus muscle.

Each rat participated in the four series in the following order: pre-mapping 1, pre-mapping 2, novel mapping and post-mapping. Each session was started immediately after the previous session was completed. In the pre-mappings 1 and 2 and in the post-mapping, the motor map was estimated by the conventional mapping procedure. In the novel mapping the stimulation was not applied to the same electrode within 2 s, because motor cortical stimulation using the same electrode at a rate >1 Hz could alter motor representation and excitability (Nudo et al., 1990). Given that the interstimulus interval in the novel mapping is 4 Hz, the MT was estimated for more than 1 electrode at a given time. This also meant that, as the mapping progressed and the MT was determined for most of the 32 electrodes, pauses were injected into the stimulation sequence. The series of four sessions was repeated twice to estimate the ECR and soleus motor maps separately. In six rats the ECR motor map was estimated before the soleus motor map; the order was reversed in the remaining six rats. All the mapping procedures were conducted during periods of stable anesthesia in which weak spontaneous vibrissae movements were observed (Friedberg et al., 1999; Tandon et al., 2008) and halted during occasional periods of shallow or deep anesthesia. The level of anesthesia was designed to be “deep” when no EMG response was observed to the 1.0 mA stimulation in all stimulating electrodes or if heart rate was slower than 280 beats/min. If rats were under “shallow” anesthesia (spontaneous movements in the limbs or heart rate >350 beats/min), 10 mg of ketamine was administered intramuscularly (Frost et al., 2013). After the ECR and soleus mappings, we measured the coordinates of the bregma and of the four stimulating electrodes located at the corners of the μECoG array by using a stereotaxic micromanipulator (SM-11; Narishige). These coordinates were then used to calculate the position of the electrodes.

#### Data analysis

We calculated Spearman's correlation coefficient and intraclass correlation coefficient (ICC) for the three different pairs of motor maps: (1) between motor maps assessed in the pre-mapping 1 and 2 to obtain a baseline correlation value; (2) between motor maps assessed in the pre-mapping 1 and the novel mapping to validate the novel mapping method; and (3) between motor maps assessed in the pre-mapping 1 and the post-mapping to examine whether the 4-Hz CSS mapping procedure had altered motor representations. ICC was calculated using two motor map parameters with reference to human preclinical research: minimum MT (Ngomo et al., 2012) and map area (Uy et al., 2002). Minimum MT represents the cortical excitability. As map area (the size of the motor map) we considered the total number of responsive electrodes (MT ≦ 0.95 mA). ICC is an index of test-retest reliability (McGraw and Wong, 1996) and values ≧0.8 are thought to be very reliable (Landis and Koch, 1977).

Spearman's correlation was calculated with the ranks of the MTs for each map. We performed 2,000 random resamplings of the ranks to generate the distribution that was expected when the relationship of two motor maps is random. The original correlation coefficient exceeding the 99th percentile of the empirical distribution was deemed to be significant. In addition, the Spearman's correlation coefficients calculated with the three different pairs of the motor maps were compared using Friedman test with “Pair of Mapping Sessions” as a within-subject factor. Time durations required to map the cortex was also compared using Friedman test with “Mapping Sessions” as a within-subject factor. *Post-hoc* analysis was conducted by Wilcoxon test with Bonferroni correction. The level of significance was set at α = 0.01.

### Comparison of CSS and ICMS mappings

#### Experimental procedure

The reliability of CSS mapping has already been proved with respect to ICMS (Molina-Luna et al., 2007). However, for further validation we performed ICMS mapping on two of the 12 rats that were submitted to the CSS mapping and compared the motor maps assessed by CSS with those assessed by ICMS. After the above-described CSS mapping (see Experimental Procedure section), we again examined both the ECR and soleus motor maps by the novel CSS mapping method. In one rat the ECR motor mapping was performed first. The order was reversed for the next rat. We performed both the ECR and soleus motor mappings twice because the size of the μECoG array was not sufficient to cover the entire motor cortex. After each mapping procedure, the coordinates of the bregma and of the four electrodes located at the corners of the μECoG array were measured.

ICMS was performed using an epoxy-insulated tungsten microelectrode with an impedance of 0.1–1 MΩ (FHC, Bowdoin, ME). A copper plate (5 × 10 cm^2^) covered with a saline-soaked gauze was put under the rat's abdomen for stimulus ground. The stimulus consisted of ten 200-μs cathodal pulses delivered at 125 Hz. The train was repeated at 1 Hz. The tungsten electrode was positioned using a 3D manipulator (SM-11; Narishige). The electrode was advanced to a depth of 1500–2000 μm perpendicular to the cortical surface, with an interpenetration distance of 500 μm. The stimulation was started at 60 μA and progressively reduced to assess the ECR and/or soleus MT. If no movements were evoked at 60 μA, ICMS was halted and the site was marked as nonresponsive. ICMS mapping continued until both the ECR and soleus motor areas were surrounded by nonresponsive sites.

#### Data analysis

Similarities between the motor maps identified by the novel CSS and ICMS mappings were assessed by the Spearman's correlation coefficient, calculated as follows:

$$r=\frac{\sum_{n}\left[R_{\text{MTicms}}\left(x,y\right)-\frac{n+1}{2}\right]\sum_{n}\left[R_{\text{MTcss}}\left(x,y\right)-\frac{n+1}{2}\right]}{\sqrt{{\sum_{n}\left[R_{\text{MTicms}}\left(x,y\right)-\frac{n+1}{2}\right]}^{2}{\sum_{n}\left[R_{\text{MTcss}}\left(x,y\right)-\frac{n+1}{2}\right]}^{2}}}$$

*x* and *y* represent coordinates where the ECR or soleus movements were evoked by the ICMS; *n* represents the number of responsive sites in the ICMS mapping. *R*_MTicms_ is the rank of the MT among the responsive sites in the ICMS mapping. *MT*_CSS_ represents estimated MT in the CSS mapping, which was extracted from the linearly interpolated motor map (**Figure 4**). *R*_MTCSS_ is therefore the rank of the estimated MTs at locations (*x, y*) that were responsive sites in the ICMS mapping. We then performed 2000 random resamplings of the ranks to generate a distribution that was expected when the relationship of the two motor maps is random. The original correlation value exceeding the 99th percentile of the empirical distribution was deemed to be significant.

### Electrochemical impedance spectroscopy

Advantages of PEDOT-CNT coated μECoG electrodes are low electrode impedance and a large charge injection capacity (Castagnola et al., 2013). This enabled us to perform both field potential recordings and CSS. However, the electrode arrays could degrade and fail to function correctly with repeated charge injection. Thus, we tested the stability of the electrochemical impedance spectra before and after the repeated CSS mapping procedure.

Impedance spectra of the 32 μECoG electrodes were measured before PEDOT-CNT coating, just after PEDOT-CNT coating (but before any current injection applied) and after the repeated CSS mappings. Here, the mapping methods followed the procedure described in section Experimental Procedure, which repeated a series of four mapping sessions twice to estimate the ECR and soleus motor maps separately. The impedance measurements were conducted by galvanostatic electrochemical impedance spectroscopy (PARSTAT 2273; Princeton Applied Research, Oak Ridge, TN) performed in saline (0.9% NaCl) by applying a current (sine wave) of 300 nA (root mean square) at 10 frequencies per decade over the range of 1 Hz–10 kHz. The galvanostatic device was connected to a three-electrode electrochemical cell with a platinum counter electrode and an Ag/AgCl reference electrode. The stability of electrode impedance was tested by an ANOVA with “Frequency” and “Electrode Conditions” (i.e., before PEDOT-CNT coating, just after PEDOT-CNT coating and after the CSS mappings) as within subject factors and by *post-hoc* analyses using a paired *t*-test with Bonferroni correction. All data analyses and statistical tests were performed using MATLAB 2014a (The MathWorks, Natick, MA).

### Somatosensory evoked potential recordings

Electrochemical impedance spectroscopy provides evidence of electrical stability, but is not sufficient to validate whether the electrodes to which electric current has been repetitively applied can be used for field potential recordings. We therefore recorded somatosensory evoked potentials (SEPs) in one of the rats not involved in the ICMS mapping. After conducting the CSS mappings described above and while the rat was under anesthesia, an incision on the skin was made and the left brachial plexus and the left sciatic nerve were clipped by two pairs of enamel-insulated silver electrodes (400 μm diameter, 2–3 mm separation). A μECoG array, which was utilized for the CSS mappings in the same rat, was repositioned over the right somatosensory cortex. Each nerve was separately stimulated 200 times consecutively via the bipolar electrodes (2.0 ms, rectangular pulse, frequency 1 Hz). Stimulus intensities of the brachial plexus (0.3 mA) and sciatic nerve (0.5 mA) were adjusted to be slightly above the threshold to evoke a visible twitch of the wrist and ankle, respectively. Epidural field potentials recorded from the 32 channels were band-pass filtered (3–2000 Hz with 2nd order Butterworth) and digitized at 5 kHz (Digital lynx; Neuralynx, Bozeman, MT).

## Results

### Motor maps assessed by using epidural cortical stimulation

**Figure 3:**
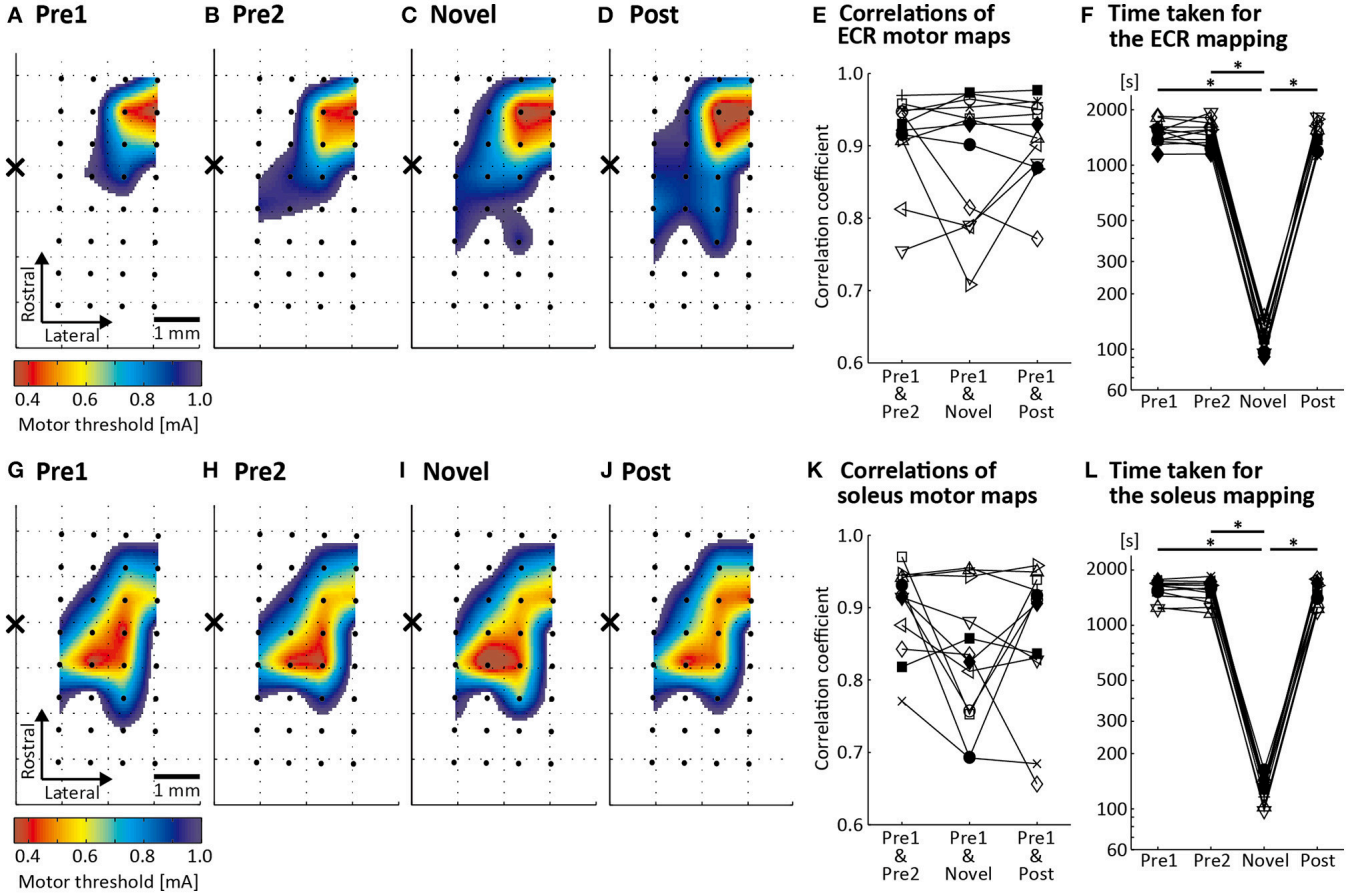
Results of motor mapping by cortical surface stimulation (CSS). Topographical spatially interpolated ECR **(A–D)** and soleus **(G–J)** motor maps from one representative rat. The color scale reflects the MT. Black dots and the cross in each panel indicate the location of the 32 stimulating electrodes and of the bregma, respectively. Motor maps of Pre1, Pre2, and Post conditions were assessed using the conventional CSS mapping procedure (conventional MT criterion and stimulus frequency of 1 Hz). Motor map of the Novel condition was assessed using the novel CSS mapping procedure (newly proposed MT criterion and stimuli repeated at 4 Hz). **(E,K)** Spearman's correlation coefficient calculated for three different pairs of motor maps in 12 rats: between the Pre1 and Pre2 conditions, between the Pre1 and Novel conditions and between the Pre1 and Post conditions. **(F,L)** Time taken for the mapping procedure in the 12 animals in the four conditions. Each line represents one animal. ^*^p < 0.01.

Figures 3A–D show the ECR motor maps from an individual rat assessed by the conventional CSS mapping, which uses the conventional MT criterion (Rossini et al., 1994) with stimulus repetition at 1 Hz (Figures 3A,B,D), and by the novel CSS mapping, which uses the newly proposed MT criterion with stimulus frequency of 4 Hz (Figure 3C). Maps were acquired in the same order as that in the figure (Figures 3A–D). A permutation test revealed that the Spearman's correlation coefficient between the motor maps assessed in the pre-mappings 1 and 2, which reflects a baseline correlation, was significant in all 12 rats (*p* < 0.01). The correlation coefficients between motor maps assessed in the pre-mapping 1 and the novel mapping and the post-mapping were also significant in all 12 rats (*p* < 0.01). There was no significant difference in the correlation coefficients depending on “Pair of Mapping Sessions” [$\chi_{\left(2\right)}^{2}$ = 0.500, *p* = 0.779: Figure 3E]. We found a significant main effect of “Mapping Sessions” on the time taken for the mapping [$\chi_{\left(3\right)}^{2}$ = 22.0, *p* < 0.001: Figure 3F]. Its median was 1506 s in the pre-mapping 1, 1504 s in the pre-mapping 2, 117 s in the novel mapping, and 1446 s in the post-mapping. *Post-hoc* analysis demonstrated that the mapping time was significantly shorter in the novel mapping than in the pre-mappings 1 and 2 and the post-mapping (*p* < 0.01). The number of required stimuli for the novel mapping ranged from 229 to 409.

Figures 3G–J illustrate the soleus motor maps of a representative rat assessed by the conventional CSS mapping (Figures 3G,H,J) and by the novel CSS mapping (Figure 3I). The maps were acquired in the same order as that in the figure (Figures 3G–J). The correlation coefficients between motor maps assessed in the pre-mapping 1 and pre-mapping 2, the novel mapping and the post-mapping were significant in all rats (*p* < 0.01). We found no significant difference in the correlation coefficients depending on “Pair of Mapping Sessions” [$\chi_{\left(2\right)}^{2}$ = 4.500, *p* = 0.105: Figure 3K]. However, there was a significant main effect of “Mapping Sessions” on the time taken for the mapping [$\chi_{\left(3\right)}^{2}$ = 23.7, *p* < 0.001: Figure 3L]. The median of the mapping time was 1646 s in the pre-mapping 1, 1557 s in the pre-mapping 2, 132 s in the novel mapping, and 1548 s in the post-mapping. *Post-hoc* test revealed that the mapping time was significantly shorter in the novel mapping than in the pre-mappings 1 and 2 and the post-mapping (*p* < 0.01). The number of required stimuli for the novel mapping ranged from 272 to 477.

ICCs for minimum MT exceeded 0.8 in all comparisons (Table 1), suggesting that cortical excitability was little fluctuating across mapping sessions and that the novel CSS mapping did not alter excitability. ICCs for map area also exceeded 0.8 in all comparisons (Table 1). This indicated that the total number of effective electrodes (MT ≦ 0.95 mA), depending on the size of the motor map, did not vary across mapping sessions.

**Table 1:** Intra-class correlation representing the test-retest variability of the motor map parameters.

**Parameter**	**Conditions**	**ICC**
ECR, minimum MT	Pre1–Pre2	0.94
	Pre1–Novel	0.89
	Pre1–Post	0.96
ECR, map area	Pre1–Pre2	0.91
	Pre1–Novel	0.83
	Pre1–Post	0.84
Soleus, minimum MT	Pre1–Pre2	0.96
	Pre1–Novel	0.85
	Pre1–Post	0.85
Soleus, map area	Pre1–Pre2	0.92
	Pre1–Novel	0.86
	Pre1–Post	0.82

*Motor maps in the conditions of Pre1, Pre2, and Post were assessed using the conventional MT criterion with the stimulus repeated at 1 Hz. Motor map in the Novel condition was assessed using the newly proposed MT criterion with the stimulus repeated at 4 Hz. Experiments were performed in the order of Pre1, Pre2, Novel, and Post*.

### Comparison of CSS and ICMS mappings

**Figure 4:**
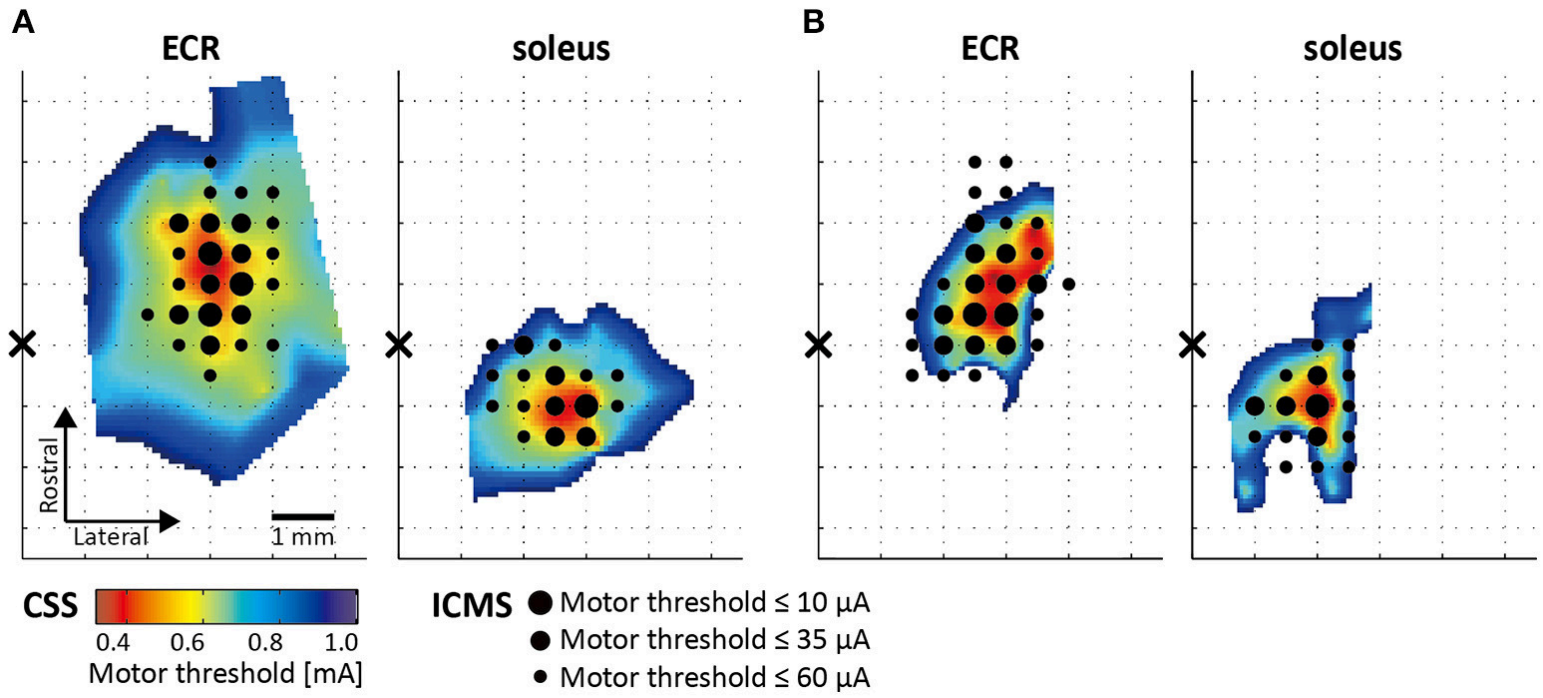
Motor maps assessed using CSS and intracortical microstimulation (ICMS). Topographical spatially interpolated motor maps obtained from two rats **(A,B)**. Color scale reflects the MT assessed by CSS, black dot size reflects the MT assessed by ICMS. Black cross represents the position of the bregma.

The ECR and soleus topographical motor maps of two rats that were assessed using the novel CSS and ICMS mapping procedures are presented in Figure 4. The color scale reflects the MT. Since the CSS mapping procedure was performed twice at different locations over the motor cortex, we merged and linearly interpolated the MTs of 64 electrode positions into a single map. The results from the ICMS motor mapping are indicated by the black dots. Dots size reflects the MT for each responsive location. The correlation between motor maps assessed by the CSS and ICMS mappings was 0.71 (Figure 4A, ECR), 0.69 (Figure 4A, soleus), 0.74 (Figure 4B, ECR), and 0.77 (Figure 4B, soleus). The permutation test showed that all four correlation coefficients were statistically significant (*p* < 0.01).

### Electrochemical impedance spectroscopy

**Figure 5:**
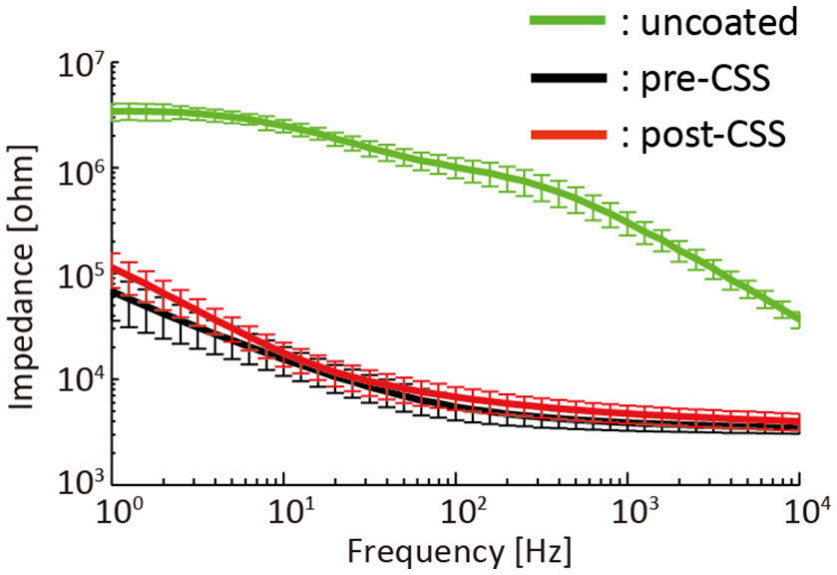
Impedance spectra of 32 μECoG electrodes (mean ± standard deviation) that were measured before PEDOT-CNT coating (uncoated), after PEDOT-CNT coating but before any current injected (pre-CSS) and after the repeated CSS mappings (post-CSS).

Figure 5 illustrated the impedance spectra of the 32 μECoG electrodes that were measured before PEDOT-CNT coating (uncoated), after PEDOT-CNT coating but before any current injected (pre-CSS) and after repeated CSS mappings (post-CSS). The total number of stimuli applied to the 32 electrodes was 8788, which was approximately 25 times higher than the average number of stimuli required for the novel mapping. ANOVA applied to the impedance spectra showed a significant main effect of “Frequency” [*F*_(40, 1240)_ = 965, *p* < 0.001] and “Electrode Conditions” [*F*_(2, 62)_ = 1599, *p* < 0.001] and a significant interaction of “Frequency” × “Electrode Conditions” [*F*_(80, 2480)_ = 930, *p* < 0.001]. The *post-hoc* test revealed that the impedance spectra of the uncoated electrodes were significantly higher than the pre- and post-CSS electrodes for all the frequency ranges (*p* < 0.001). The averaged impedance below 250 Hz, which is generally the upper limit of the gamma band in the ECoG analysis, was more than 20 times higher for the uncoated electrodes than the pre- and post-CSS electrodes. The mean impedance spectra of the post-CSS electrodes were significantly higher than those for the pre-CSS electrodes for all the frequency ranges except between 10–50 Hz (*p* < 0.05), but the difference was not more than twice.

### Somatosensory evoked potential recordings

**Figure 6:**
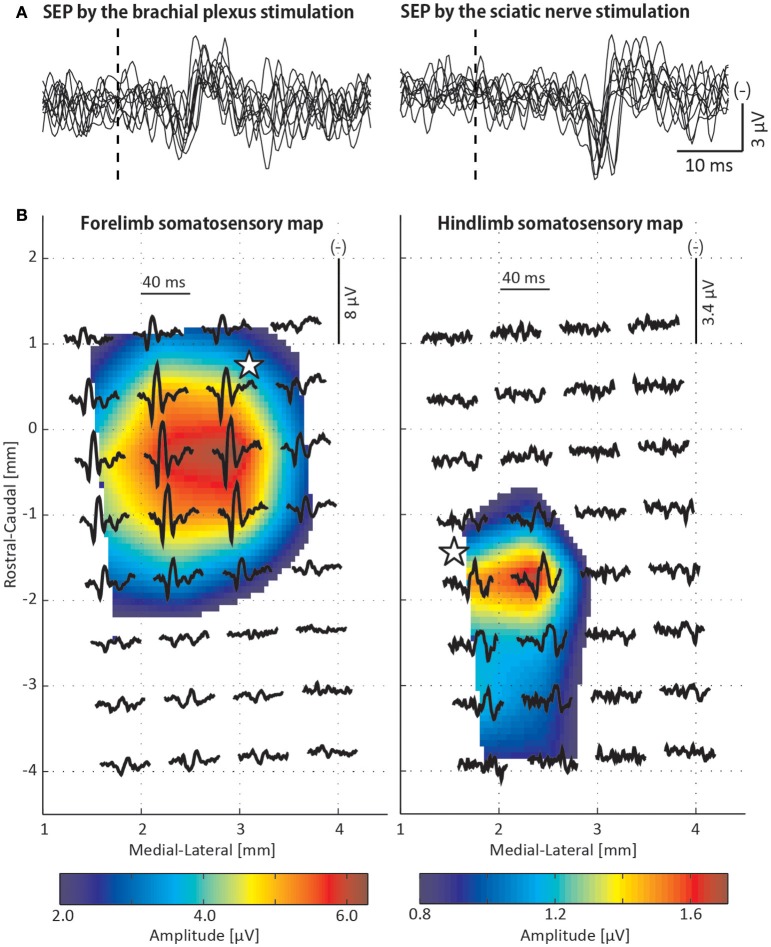
Somatosensory evoked potential (SEP) recordings. **(A)** Ten overlaid SEP traces recorded from a single μECoG electrode that was repeatedly used for cortical stimulation. Vertical dotted line reflects the timing of the peripheral nerve stimulation. **(B)** Forelimb and hindlimb somatosensory maps that were composed of the SEPs elicited by the stimulations to the brachial plexus and sciatic nerve, respectively. Waveforms represent the average of 200 SEP traces recorded from each electrode. A somatosensory map was constructed using peak-to-peak amplitudes between initial positive and negative peaks of the SEP and spatially interpolated. The white star is the ECR/soleus motor hotspot where the lowest MT was observed in Figures 3A,G. The origin of the map corresponds to the position of the bregma.

The stimulation of both the brachial plexus and the sciatic nerve induced short latency P1 (first positive peak) and N1 (first negative peak) components (Figure 6A). The latency of the P1 and the N1 components was shorter for the brachial plexus stimulation (P1, 10.0 ms; N1, 14.8 ms) than for the sciatic nerve stimulation (P1, 19.2 ms; N1, 25.1 ms). The somatosensory maps were composed of the P1-N1 amplitude of the SEP (Figure 6B). The hotspot where the largest P1-N1 peak-to-peak amplitude was observed varied between the forelimb and the hindlimb maps (forelimb: 2.9 mm lateral and 0.3 mm caudal to the bregma, hindlimb: 2.4 mm lateral and 1.7 mm caudal to the bregma).

## Discussion

In the present study, we introduced an automatic, less-invasive, motor mapping procedure and a new algorithm that requires a smaller number of stimuli for MT estimation. The reliability of the novel CSS mapping procedure, which uses the newly proposed MT algorithm and a 4-Hz stimulation frequency, was validated in 12 male rats. The results suggest that a motor cortical region could be identified in less than 3 min by using this novel mapping method. The time duration was one-twelfth shorter than that of the conventional mapping procedure performed using a 1-Hz stimulation frequency and the traditional MT criteria (Rossini et al., 1994). The novel procedure we described here showed equally high reliability with respect to the more traditional one. Finally, we found significant correlations between the motor maps assessed by the novel CSS and those acquired by ICMS, providing further evidence for the reliability of our novel CSS mapping.

### Reliability of the novel motor threshold (MT) and high-frequency stimulation mapping

The present study proposed a new algorithm for relative frequency MT estimation (Figure 2B) in which the next stimulus intensity was decreased or increased by 5% of the MSO (0.05 mA) if an MEP response elicited by the actual stimulus was larger or smaller than the predetermined amplitude. This procedure is similar to the threshold tracking techniques used to estimate human peripheral nerve excitability (Bostock et al., 1998). In this approach, one first sets a target response level and then the stimulus is automatically stepped up or down depending on whether the previous response was smaller or greater than the target one. However, since this was originally proposed for online tracking of changes in excitability, we extended this idea by adding a stopping rule for estimating the MT. If the intensity change in the last five stimuli was within ±5% of the MSO, the MT was defined as the average stimulus intensity of the last two stimuli. The reliability of this newly proposed MT criterion was tested in comparison with the conventional relative frequency MT (Rossini et al., 1994), which is used for most human preclinical studies (Groppa et al., 2012). The conventional criterion defines the MT as the lowest stimulus intensity capable of evoking MEPs greater than the predetermined amplitude in at least five of 10 consecutive trials. In the results, we found that motor maps estimated using the conventional MT and the newly proposed MT were significantly correlated. Moreover, this correlation was not statistically different from the correlation between two motor maps both estimated by the conventional MT. ICC of the map area showed high test-retest reliability in all comparisons. This means that a stable MT estimation is feasible with the stopping rule newly proposed in the present study.

We performed motor mapping four times. The first, the second and the fourth mappings were conducted using the conventional MT estimation algorithm with biphasic CSS at 1 Hz, and the third mapping was conducted using the novel MT estimation algorithm with biphasic CSS at 4 Hz. One can argue that there might be plasticity-dependent effects after 4-Hz CSS, as repeated stimulation to the M1 is known to induce tonic effects (Nudo et al., 1990; Di Lazzaro et al., 2002; Quartarone et al., 2005). However, the ICC of the minimum MT calculated before and after the 4-Hz mapping showed high reliability, indicating that cortical excitability was stable over time with no effects of the 4-Hz mapping procedure on the modulation of motor maps. Herein, what could explain the differences between our null results and previously shown tonic effects of the repetitive cortical stimulation? First, we utilized biphasic stimulus pulses, which are known to affect plasticity less than monophasic stimulus pulses (Nakamura et al., 2016). In addition, the number of stimuli in our novel mapping procedure was much smaller than the number of stimuli known to significantly affect plasticity. A human study using biphasic pulses at 90% of the MT intensity revealed that cortical excitability is modulated following 900 consecutive stimuli at 5 Hz but not following 600 stimuli (Quartarone et al., 2005). It has also been shown that more than 10 consecutive supra-MT stimuli to the motor cortex repeated at 5 Hz enhanced cortical excitability (Di Lazzaro et al., 2002), but this rarely happened in our short mapping procedure because in our procedure the next stimulus intensity was below the MT if the previous stimulus was able to induce a detectable MEP.

Although, our mapping algorithm avoided applying stimulation through the same electrode within 2 s, it did not restrict the stimulation of an electrode close to it. Since ICMS is known to evoke both inhibitory and facilitatory responses up to 2 mm away from the stimulation site (Baker et al., 1998), the MT assessed at an electrode location close to the previously stimulated site could be either over- or under-estimated. We considered this as a possible cause for the low (approximately <0.8, but statistically significant) correlation coefficients observed in several animals (Figures 3E,K). However, we are aware that spatial and temporal effects of dual-site CSS on cortical excitability needs to be investigated in future studies.

It should be pointed out that there are different procedures for determining the MT. A traditional procedure to estimate the MT is the relative frequency method, first proposed by Rossini et al. (1994). Our results revealed that MT estimated by our newly proposed relative frequency method was equally reliable as the traditional method, although the number of stimuli was approximately one-fourth the amount in the novel criterion. However, such relative frequency MT estimation has been criticized because it neglects the probabilistic nature of the MT, and thus, might be too susceptible to variations in responses to cortical stimulation (Groppa et al., 2012). In humans, the most accurate and robust MT is provided by adaptive MT estimation that uses a cumulative Gaussian distribution function to model a relationship between the stimulus intensity and the probability of eliciting a muscle twitch at rest (Awiszus, 2003; Mishory et al., 2004). However, the adaptive method requires a priori assumption of the threshold spread (i.e., corresponds to the standard deviation of Gaussian distribution), which is standardized for human TMS at rest, but not for human in motor tasks or animals. Note that the properties of neural excitability could be different among different species and contexts. Given these practical limitations of the adaptive method, our new criterion for relative frequency MT estimation could be a good candidate that is applicable in most situations, not only invasive stimulations (e.g., ICMS) in rodents and primates but also non-invasive stimulations (e.g., TMS) in human experiments.

### Advantages of cortical surface stimulation (CSS) mapping with μECoG electrodes

The advantages of cortical mapping using an array of surface electrodes are the minimal invasiveness and the simplification of the experimental procedures. A previous study using an epidural array for motor mapping did not show motor deficits and spared integrity of cortical tissue (Molina-Luna et al., 2007). Such minimal invasiveness is preferable when aiming at longitudinal assessment of cortical plasticity or at combination with neural activity measurements. In the latter case, CSS mapping can be used as a tool to localize a functionally relevant cortical motor region where the neural activity will then be assessed, without damaging the neurons. Besides its minimal invasiveness, CSS mapping also does not require to move the electrodes. It is not as skill demanding as ICMS. Moreover, recording of field potentials from the electrodes that have been used for the stimulation is an advantage when using surface electrodes. In this study, we tested the impedance of the μECoG electrodes affecting the signal-to-noise ratio before and after the repeated CSS mapping procedure and confirmed that it remained far lower than that of the nanomaterial-uncoated electrodes. The result from the SEP recordings provided evidence that the electrodes to which electric current is repetitively applied can be used for field potential recordings. If a system allows simultaneous stimulation and recording, μECoG electrodes will let to perform concurrent mappings of M1 representation and its effective connectivity by recording cortical stimulation-evoked potentials from the electrodes surrounding a stimulus electrode to assess direct causal influences within and between cortical regions (Keller et al., 2014). The recordings of cortical evoked potentials would be of interest for mapping areas that do not provide peripheral (i.e., muscle twitch) responses to the cortical stimulation but where antidromic cortical responses are provoked by the stimulation to M1, such as the supplementary motor area (Matsumoto et al., 2007). However, since the cortical stimulation provokes a large electrical artifact, which obscures the cortical response in the first millisecond after stimulation, removing this artifact remains a challenge (Tomasevic et al., 2017).

One disadvantage of CSS is that the area where cortical stimulation can be applied is limited by the size of the electrode array. The present study utilized 32 electrodes arranged on an area of 5.0 × 2.2 mm^2^. This size was sufficient to identify hand/foot motor region in rats, but not to cover the entire hand/foot area. In this sense, ICMS can determine complete motor maps that cover the whole region of interests. In addition, CSS requires higher currents to ignite cortical neurons than those required by ICMS, because the surface electrode is at further distance from the target neurons than the ICMS electrode, the tip of which is located in the cortical layer V. Higher currents spread to larger volume of tissue and may result in less focal stimulation and overestimation of the map size (Molina-Luna et al., 2007). Thus, we performed ICMS mapping in two of the 12 rats for additional validation of the novel CSS mapping. The results showed statistically significant correlations between the CSS and ICMS maps in both rats, indicating that the CSS validly identified motor representation in comparison with the ICMS mapping.

### Benefits and possible applications of the automatic motor mapping system

In many studies electrophysiologically investigating motor representation in animals, the mapping procedure includes multiple manual/observational processes. Muscle responses to the stimulation are often determined by visual observation (Ramanathan et al., 2006; Bashir et al., 2012; Frost et al., 2013; Rouse and Schieber, 2016). Location and intensity of the stimulation are also manually selected and adjusted. The process of MT determination, such as the method to select the next stimulus intensity and criteria for completing the MT estimation, is usually investigator-dependent. Our motor mapping system automatically calculates MEP amplitude of a target muscle and estimates the MT in each stimulus electrode using the novel relative frequency algorithm that was fully investigator-independent.

We argue that the benefits of our automatic motor mapping procedure are two fold. First, time for motor mapping is shortened to the order of a few minutes. This may shed light on the significance of short-term reorganization of motor representations during motor learning and functional recovery. Recently, structural modifications induced by learning tasks were suggested to occur in the order of minutes to hours (Hofstetter et al., 2016). Automatic mapping will bridge the gap between functional and structural plasticity in short time scales. Furthermore, brief experimental procedures minimize the confounding effects of time on the mapping results and thus can highlight plasticity-inducing situations, such as rehabilitation after brain injury.

Second, theoretically, the automatic motor mapping system can be used with any type of stimuli, such as intracortical microelectrodes and micro-optical fibers, because our new criterion for relative frequency MT estimation does not depend on specific properties of neural excitation that would vary among species and types of stimulation. This will allow us to detect dynamic plasticity occurring in micro-neural structures as well. Moreover, although our automatic mapping system was tested with 32 electrodes, the system and the novel MT algorithm can be extended to any number of electrodes. Increasing the number of the electrodes (i.e., enlarging the area where CSS is applied by a single electrode sheet) will enable researchers to cover several motor representations at once.

### Practical considerations

First, it remains unknown whether our epidural mapping procedure could be applied as-is to subdural approach as well. A previous study revealed that the resolution of epidural and subdural current spread is almost identical in rats, but not in humans (Slutzky et al., 2010). This implies our epidural mapping procedure could be used for a subdural approach in animals with thin dura, such as rodents and marmosets (Tia et al., 2017), but not in macaques and humans that have a thick dura, without conducting follow-up studies.

Second, our stimulation parameters (10 ms stimulus trains consisting of 0.5 ms biphasic pulses delivered at 1000 Hz) were quite different from those generally used for direct electrical stimulation mapping in humans (3–20 s stimulus trains consisting of 0.2–1 ms biphasic pulses delivered at 50–60 Hz; Picht et al., 2013; Krieg et al., 2014). Thus, for clinical translation, future studies that systematically compare the effectiveness and safety of stimulus frequencies and duration that electrically stimulate the brain will be required in humans. Note that the intrinsic property of the electric field distribution and resulting after-discharges of neurons, caused by the brain stimulation, could vary between rodents and humans.

Third, although we carefully monitored the rodents during mapping experiments to maintain stable anesthetization, the level of anesthesia is known as a factor that can significantly affect the response to stimulations (Mégevand et al., 2008; Tandon et al., 2008) and could have changed without accompanying behavioral observations. Thus, it is desirable to avoid anesthesia for the period of mapping in such experiments. Our novel mapping procedure that can shorten the time for which an animal needs to remain at rest could contribute to simplify the evaluation of motor representations under awake conditions.

## Conclusions

We established an automatic, faster, less-invasive motor mapping system and a new algorithm that requires a smaller number of stimuli for MT estimation. As a proof of concept, we tested reliability of the novel mapping procedure in 12 male rats with respect to the conventional mapping technique. The results showed that, in all tested rats, our novel CSS mapping procedure identified a motor cortical region within 3 min. This time was one-twelfth shorter than that taken in the conventional mapping method, while the reliability of both the mapping methods was equally very high. The novel CSS mapping technique may contribute to unveiling the functional significance of short-term plasticity processes in motor learning and recovery from brain injuries. Besides this advantage, particularly in the case of human patients or experimental animals that are less trained to remain at rest, reduction in mapping time will be less physically and mentally demanding and might allow us to evaluate motor maps in awake individuals as well. Furthermore, because of the minimal invasiveness, the novel CSS mapping technique can be used as a tool to localize a motor cortical region where the neural activity will then be assessed, without damaging the neurons.

## Author contributions

MTm, DR, LF, AI, and JU designed the experiment. MTm, EC, AA, and MTk performed the study. All authors were involved in drafting the work and approved it for publication.

### Conflict of interest statement

MTm, AI and JU have filed an U.S. and Japanese patent application (14/821,117 and 2014-229524, respectively) entitled “Threshold estimation apparatus, threshold estimation method, and non-transitory computer-readable information recording medium.” AI is the President and CEO of Rikaenalysis Corporation (RIKEN Venture). The other authors declare that the research was conducted in the absence of any commercial or financial relationships that could be construed as a potential conflict of interest.
